# Single‐Cell Level Characterization of B Cell Depletion and Repopulation Following Rituximab in Systemic Lupus Erythematosus

**DOI:** 10.1002/art.70116

**Published:** 2026-04-20

**Authors:** Haerin Jang, Norzawani Buang, Catherine Sutherland, Wanseon Lee, Lauren Overend, Tarran S. Rupall, Katie L. Burnham, Matthew C. Pickering, Marina Botto, Emma E. Davenport

**Affiliations:** ^1^ Wellcome Sanger Institute, Wellcome Genome Campus Hinxton United Kingdom; ^2^ Department of Immunology and Inflammation Imperial College London London United Kingdom; ^3^ Centre for Human Genetics University of Oxford Oxford United Kingdom

## Abstract

**Objective:**

Rituximab, a CD20^+^ B cell depletion therapy, is frequently used to treat systemic lupus erythematosus (SLE). However, variability in patient response highlights the need for a deeper understanding of the underlying immune cell dynamics of B cell depletion and repopulation.

**Methods:**

We conducted longitudinal single‐cell profiling of nine patients with SLE treated with rituximab from pretreatment to up to 15 months post‐treatment. These were compared with eight healthy controls. We profiled 169,513 immune cells via single‐cell RNA, surface protein, B cell receptor (BCR), and T cell receptor sequencing, and bulk BCR repertoire sequencing.

**Results:**

Significant depletion of naïve, memory, and age‐associated B cells was observed early post‐treatment, followed by later repopulation of mainly transitional B cells. A fraction of antigen‐experienced B cells, particularly in nonresponders, persisted through the depletion. BCR repertoire analysis revealed reduced diversity and persistent clones in antigen‐experienced cells at early post‐treatment, but these effects were not long‐lasting. Repopulated naïve B cells in rituximab responders exhibited reduced NF‐κB pathway activation, aligning with lower B cell activating factor receptor (BAFF‐R) surface protein levels. In non‐B cells, we identified 27 differentially expressed genes across seven immune cell subtypes post‐rituximab, with regulatory CD4 T cells and double negative (DN) T cells showing the most changes. Responders specifically had increased expression of genes related to cytotoxicity, major histocompatibility complex class II antigen presentation, and T cell activation in CD4 T central memory and DN T cells.

**Conclusion:**

Our longitudinal profiling provides single‐cell resolution of the shifts in immune cell dynamics following B cell depletion.

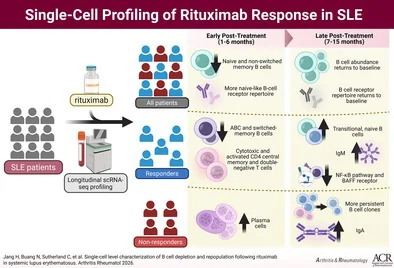

## INTRODUCTION

Systemic lupus erythematosus (SLE) is a chronic autoimmune disease characterized by widespread inflammation and tissue damage in multiple organs, including the skin, kidneys, joints, and central nervous system. Dysregulated immune responses, notably aberrant B cell activity producing autoantibodies, drive disease.^1^ Historically, treatment relied on broad immunosuppressants that often carried significant side effects. However, advances in our understanding of SLE pathophysiology have enabled targeted biologic therapies including anti‐BAFF, anti‐CD20, and anti‐interferon (IFN)‐α receptor antibodies.^2^ By focusing on specific molecular pathways and cell types involved in disease manifestation, these therapies offer targeted and potentially safer options and reduce reliance on immunosuppressants. However, our incomplete understanding of treatment mechanisms has limited success in clinical trials.

Rituximab, an anti‐CD20 monoclonal antibody, is frequently prescribed off‐label for SLE^3^ despite failing to meet endpoints in clinical trials.^4^, ^5^ It depletes circulating CD20^+^ B cells, which is followed by gradual repopulation over months. By removing B cells, rituximab likely reduces autoantibody production and proinflammatory cytokines,^6^ although its precise mechanisms are not completely understood, particularly because long‐lived plasma cells and tissue‐resident B cells are spared,^7^ yet clinical improvement is often observed. Similarly, it is unclear what underpins variability in patient response.^3^ With emerging B cell depletion therapies, such as anti‐CD19 CAR‐T therapy and obinutuzumab,^8^ understanding changes in the immune response following these treatments is increasingly important.

Previous studies have examined B cell subpopulations following rituximab using flow cytometry^9^, ^10^ and bulk transcriptomics,^11^ but molecular changes using single‐cell technologies remain unexplored. Single‐cell RNA‐seq (scRNA‐seq) reveals cell‐type–specific transcriptome changes as well as cell abundance. This approach is particularly advantageous for studying immune cells, for which gene function is highly cell‐type–specific. Studying B cell depletion therapy with scRNA‐seq offers insights into dynamic changes in not only B cell subtypes but also non‐B cells, which helps for understanding the broader immune response and potential off‐target effects. For example, a single‐cell study on CD19 CAR‐T cell mediated B cell depletion in SLE found reduced Type I interferon‐induced gene expression in monocytes and T cells but not B cells.^12^

Here, we characterized B cell depletion and repopulation following rituximab in nine patients with SLE at the single‐cell level. Using a multimodal approach comprising gene expression (scRNA‐seq), surface protein levels (cellular indexing of transcriptomes and epitopes by sequencing [CITE‐seq]), and B cell receptor (BCR) and T cell receptor (TCR) repertoire data, we analyzed changes in immune cells. Combined with bulk BCR data, we observed dynamic shifts in B cell subtypes after depletion and repopulation, whereas the BCR repertoire remained largely unchanged long‐term. By stratifying the patients into responders and nonresponders to rituximab, we found that repopulated naïve B cells in responders are transcriptionally distinct from pretreatment and observed an increase in cytotoxicity and activation related genes in central memory CD4 T cells (CD4 TCM) and double negative (DN) T cells. Our findings offer a detailed view of immune cell dynamics in patients with SLE receiving rituximab.

## MATERIALS AND METHODS

### Sample collection and sequencing

Blood samples were collected from patients with SLE who were aged at least 18 years old and receiving rituximab at the Imperial Lupus Centre, Imperial College Healthcare NHS Trust in the United Kingdom. Patients were rituximab‐naïve, except for one who received rituximab more than five years before this study. “Early post‐treatment” samples were collected 1 to 6 months after treatment, and “late post‐treatment” were collected 7 to 15 months post‐treatment to capture broad phases of B cell depletion and repopulation. Response was determined at 12 months post‐treatment following the criteria outlined in the Supplementary Methods. Healthy controls were also sampled. Participant ancestry was self‐reported from fixed categories. Samples were obtained from the Imperial College Healthcare Tissue and Biobank (ICHTB). ICHTB is supported by the National Institute for Health Research Biomedical Research Centre based at the Imperial College Healthcare NHS Trust and Imperial College London. ICHTB is approved by Wales REC3 to release human material for research (22/WA/0214), and the samples for this project (Ref: R13010‐3A) were issued from subcollection reference number IMM_MB_15_027 and IMM_MB_13_001. Written informed consent was obtained from all participants.

From all collected blood samples, single‐cell gene expression, surface proteins (n = 137), TCR, and BCR were sequenced. Peripheral blood mononuclear cells (PBMCs) were isolated and cryopreserved. Sequencing libraries were generated using the 10× Chromium platform and sequenced on Illumina NovaSeq6000. Bulk BCR sequencing followed the protocol from Bashford‐Rogers et al.^13^ Details on sequencing are in the Supplementary Methods. Flow cytometry was used to quantify B cell frequencies (CD19^+^ B cell percentage out of live CD45^+^ cells) (Supplementary Methods) for comparison with single‐cell data.

### Data preprocessing

Cell Ranger multi v7.0.0 (10× Genomics) yielded 256,696 cell‐containing droplets. Post quality control, 169,513 cells remained (Supplementary Methods and Supplementary Figure 1). Filtered BCR and TCR contigs from Cell Ranger were processed using Immcantation^14^ and IgBLAST^15^ (v1.21.0), resulting in 13,981 BCR and 67,062 TCR sequences for analysis.

Multimodal integration between scRNA‐seq and CITE‐seq data was performed with totalVI.^16^ The resulting low‐dimensional latent representations were used for neighborhood construction (k = 15) and uniform manifold approximation and projection (UMAP) generation. Unsupervised clustering used the Leiden algorithm.^17^ Cell types were annotated using canonical RNA and surface protein markers, with B cell annotation informed by isotype usage and mutation frequency (Supplementary Figures 2 and 3). Normalized CITE‐seq values from totalVI were used for surface protein level analyses, including CD20 and B cell activating factor receptor (BAFF‐R). Details on preprocessing are in the Supplementary Methods.

Bulk BCR sequencing data were processed using Immcantation and IgBLAST v1.21.0 (Supplementary Methods) resulting in 1,028,624 unique sequences. Because of technical issues, IGHV4 sequences were either not amplified or removed during post‐processing.

### Differential abundance analysis

Differentially abundant cell populations between time points were identified using MiloR (v.2.0)^18^ with a mixed effect model adjusting for individual patients. B cell subtype analyses used B cell‐only neighborhoods. Spatial false discovery rate (FDR) <0.1 was used to determine significance. Analysis methods are detailed in the Supplementary Methods.

### BCR and TCR repertoire analysis

Optimized Likelihood estimate of immunoGlobulin Amino‐acid sequences^19^ was used to calculate the generation probability of CDR3 sequences. Repertoire diversity was assessed via Shannon entropy on size‐matched samples using the *dit* Python package^20^ (Supplementary Methods). Heavy chain single‐cell and bulk BCR data were combined to identify clonal lineages across time points. Clustering of sequences into clonal groups was conducted using Change‐O.^21^ Clones present at pretreatment and one post‐treatment time point were considered persistent, whereas pretreatment‐only clones were nonpersistent. To account for varying numbers of antibody secreting cells, bulk BCR sequences were not weighted by unique molecular identifier counts.

### Differential gene expression analysis and pathway analysis

We used a pseudobulk approach, aggregating raw counts by sample and cell type, to identify differentially expressed genes (DEGs). Trimmed mean of M values (TMM) normalization was followed by a quasi‐likelihood approach from edgeR^22^ with model covariates as patient and cellular detection rate (the fraction of detected genes per cell to adjust for sequencing depth as described in Soneson et al^23^). FDR <0.05 defined significance.

Only cell types with more than 10 cells per sample were analyzed to ensure that the pseudobulk expression accurately represented the gene expression profile. For non‐B cells, we compared pretreatment with early post‐treatment. To further investigate changes in interferon‐stimulated genes, we scored cells using “scanpy.tl.score_genes” from Scanpy (v1.10.1^24^) with “Interferon signaling” pathway genes from Reactome (R‐HSA‐913531).

To identify differences in depleted and repopulated B cells, we conducted a differential gene expression analysis in naïve B cells from responders at pretreatment and late post‐treatment. Gene set enrichment analysis was performed with Reactome (ReactomePA v1.48.0, reactome.db_1.88.0^25^) using log FC‐ranked gene lists (including up‐ and down‐ regulated genes). We assessed the direction and magnitude of NF‐κB pathway activation in naïve B cells using the PROGENy.^26^ Pathway activation scores were calculated using decoupleR (v.2.8.0).^27^

To identify genes in non‐B cells where response status altered expression changes, we implemented the approach detailed in the edgeR User's Guide (Section 3.5). This identifies genes where changes in expression between time points differ depending on response. To confirm these results were robust, a permutation analysis was conducted. Detailed methods are available in the Supplementary Methods.

**Data availability statement.** Raw single‐cell RNA‐seq, CITE‐seq, and repertoire sequencing data have been deposited in the European Genome‐phenome Archive under accession EGAS00001006798. Processed single‐cell data have been deposited in Zenodo (https://zenodo.org/records/17868028). Data will be released and publicly accessible at the time of publication.

## RESULTS

### Longitudinal single‐cell profiling of rituximab response in SLE

**Figure 1:**
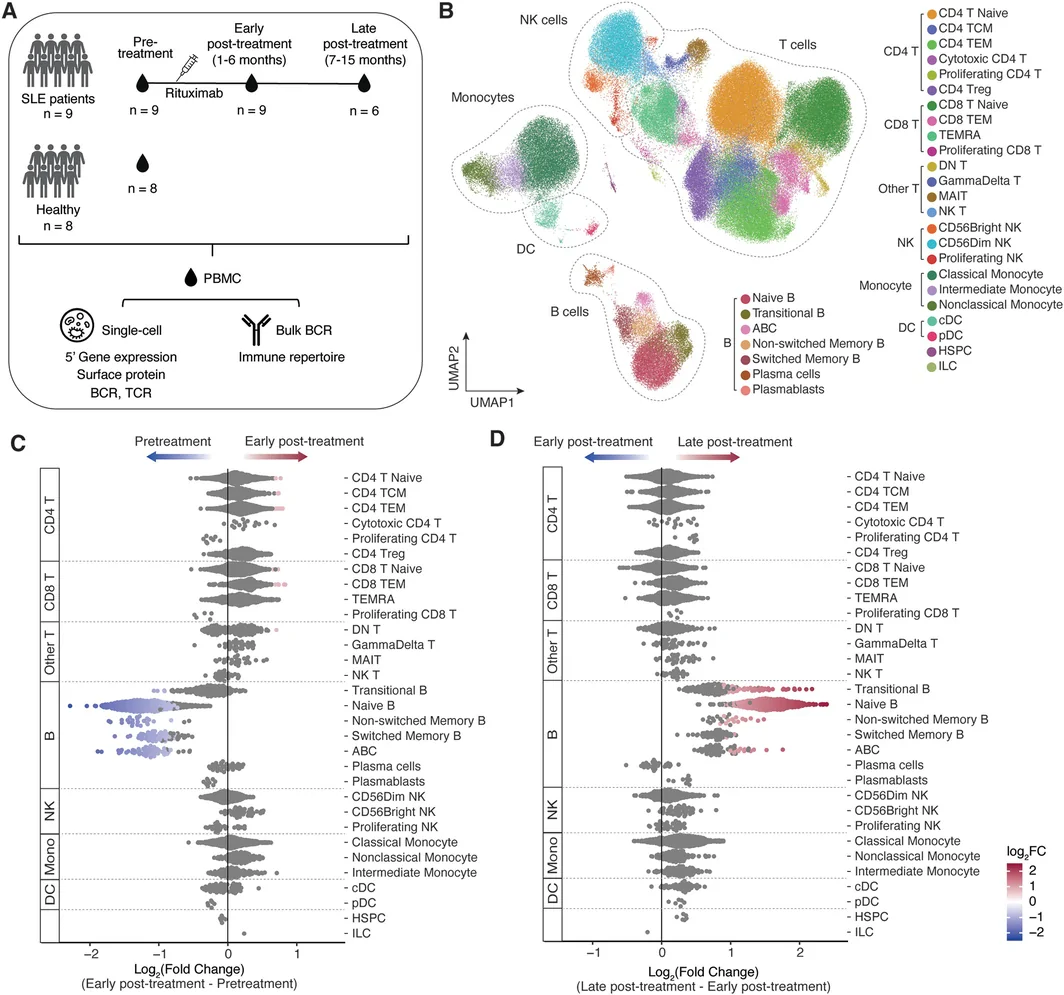
Longitudinal single‐cell profiling of response to rituximab in SLE. (A) Overview of study design. PBMCs were collected from nine patients with SLE and eight healthy controls at three time points: pretreatment, early post‐treatment, and late post‐treatment. scRNA‐seq, CITE‐seq (surface protein profiling), single‐cell BCR/TCR sequencing, and bulk BCR sequencing were performed. Pretreatment bulk BCR repertoire data were available for eight of nine patients with SLE. (B) UMAP of 169,513 cells, annotated into 31 immune cell types. Differential cell abundance between (C) pretreatment and early post‐treatment (n = 9 vs 9) and (D) early post‐treatment and late post‐treatment (n = 9 vs 6). Each point represents a cell neighborhood, where most cells belong to the annotated subtype. Neighborhoods with significantly different cell abundances between time points are colored according to their log_2_FC. BCR, B cell receptor; CITE‐seq, cellular indexing of transcriptomes and epitopes by sequencing; PBMCs, peripheral blood mononuclear cells; scRNA‐seq, single‐cell RNA sequencing; SLE, systemic lupus erythematosus; TCR, T cell receptor; UMAP, uniform manifold approximation and projection.

We conducted longitudinal single‐cell profiling in nine patients with SLE treated with rituximab to characterize immune cell dynamics following B cell depletion (Figure 1A). PBMC were collected at pretreatment and at multiple time points up to 15 months post‐treatment to capture initial B cell depletion and later repopulation. Samples were also collected from eight healthy controls to provide a reference. Participant demographics are outlined in Supplementary Table 1, and the clinical information at each time point, including the number of days from rituximab, is detailed in Supplementary Table 2.

A total of 169,513 immune cells were profiled with single‐cell RNA, surface protein, BCR, and TCR sequencing. After clustering, we annotated 31 immune cell subtypes using canonical markers (Figure 1B, Supplementary Figure 2). B cell subtype annotation was additionally informed by BCR isotype usage and mutation frequency (Supplementary Figure 3).

To assess B cell depletion following rituximab, we conducted differential cell abundance analysis between early post‐treatment and pretreatment (n = 9 vs 9) (Figure 1C). Most cell neighborhoods within naïve B cells (500 of 588 neighborhoods), non‐switched memory B cells (23 of 25 neighborhoods), switched memory B cells (54 of 72 neighborhoods) and age‐associated B cells (ABCs) (defined as CD11c^+^, Tbet^+^, CD21^low^, CD27^low^, 53 of 59 neighborhoods) were significantly less abundant at early post‐treatment (spatial FDR <0.1). Other cell types remained largely unchanged, including plasma cells and plasmablasts, aligning with their low CD20 surface protein levels (Supplementary Figure 4). Transitional B cells, despite high CD20 expression, were not less abundant at early post‐treatment. Among 156 transitional B cell neighborhoods, only 4 decreased in abundance, suggesting rapid repopulation of this cell type, given the rapid turnover and limited lifespan of this cell type^28^ or, less likely, survival of these cells despite rituximab. Comparing late post‐treatment with early post‐treatment (n = 9 vs 6) (Figure 1D), neighborhoods within transitional, naïve, non‐switched memory, and ABCs increased, suggesting that repopulation of B cells has begun in patients at these time points.

### Perturbation of the BCR repertoire after rituximab

**Figure 2:**
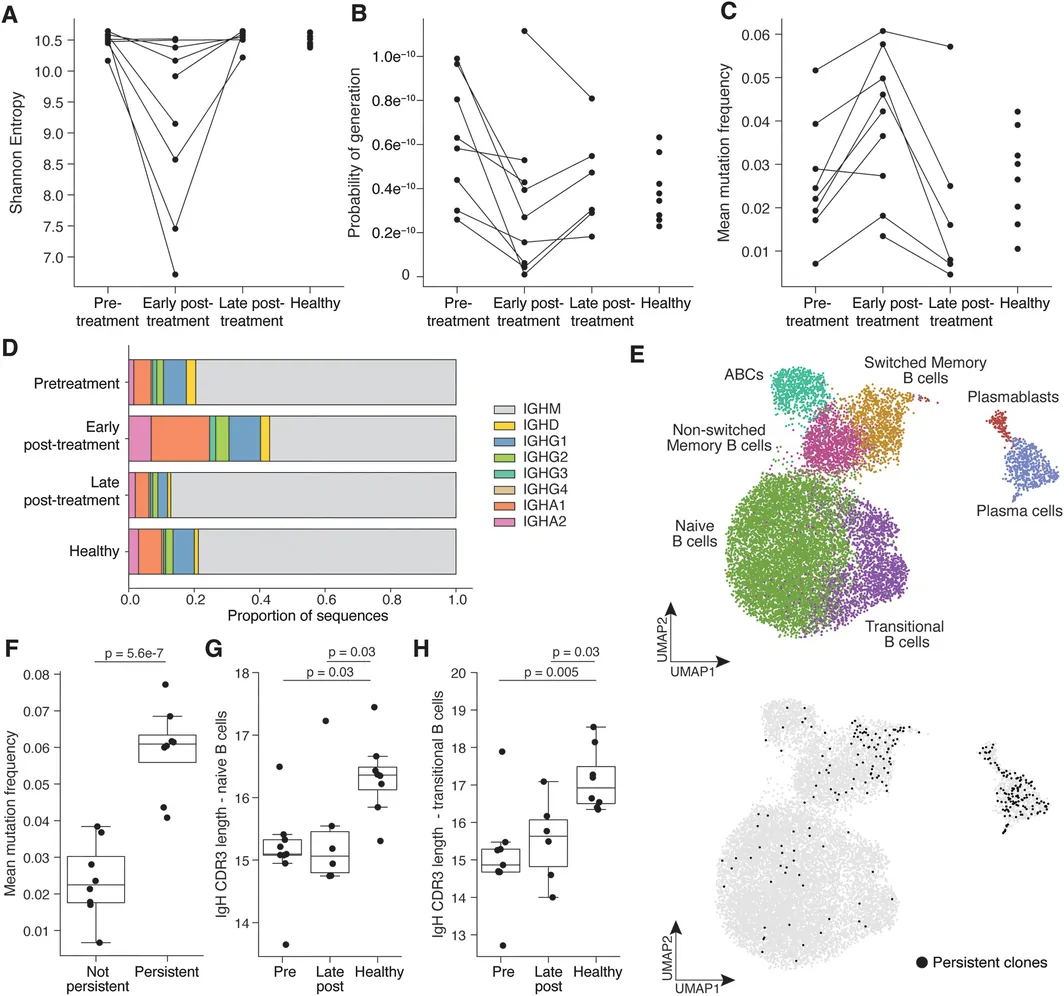
Perturbation of the BCR repertoire after rituximab. (A) Shannon entropy of bulk BCR repertoire samples across time points. Higher values indicate greater diversity. All samples were subset to 1,616 UMIs across 1,000 iterations and a mean was calculated. (B) Mean generation probability of bulk BCR repertoire samples across time points, calculated by OLGA on nucleotide CDR3 sequence. Higher generation probability indicates a sequence that is more likely to occur by chance. (C) Mean mutation frequency of bulk BCR repertoire samples across time points. (D) Isotype usage in total B cells at each time point from single‐cell data. (E) B cell UMAP highlighting cells with sequences belonging to persistent clones. Clones were considered persistent if they contained sequences from both pretreatment and one post‐treatment time point. (F) Mean mutation frequency of sequences belonging to persistent or nonpersistent clones. Paired *t*‐test. Mean IgH CDR3 length at pretreatment, late post‐treatment, and in healthy controls in (G) transitional B cells and (H) naïve B cells. FDR adjusted *P* value, Kruskal‐Wallis followed by Dunn's test. BCR, B cell receptor; CDR3, complementarity‐determining region 3; FDR, false discovery rate; OLGA, Optimized Likelihood estimate of immunoGlobulin Amino‐acid sequences; UMI, unique molecular identifier; UMAP, uniform manifold approximation and projection. Color figure can be viewed in the online issue, which is available at http://onlinelibrary.wiley.com/doi/10.1002/art.70116/abstract.

We performed both single‐cell V(D)J enrichment and bulk heavy chain BCR sequencing to profile the immune receptor repertoire pre‐ and post‐rituximab. A total of 13,981 paired BCR and 1,028,618 unique heavy chain sequences were identified. To explore repertoire characteristics, we calculated Shannon entropy, which is a measure of diversity; generation probability, which is the likelihood of a sequence being generated by random chance; and somatic hypermutation frequency (Figure 2A–C). At pretreatment, these metrics were similar across patients. At early post‐treatment, diversity and generation probability decreased in most patients, whereas mutation frequency increased. Patients with poor B cell depletion generally maintained a more diverse repertoire (Supplementary Figure 5A). By late post‐treatment, these metrics returned toward pretreatment levels. Isotype usage also varied across time points, with increased class‐switched sequences at early post‐treatment and a reduction at late post‐treatment. This is consistent with the reduction and subsequent repopulation of naïve B cells (Figure 2D).

BCR clones spanning multiple time points were observed in all patients, although the degree of overlap varied (Supplementary Figure 5B). Persistent clones were found in both responders and nonresponders, and there was minimal sharing of persistent clones between individuals. These persistent clones were enriched for switched memory B cells, plasmablasts, and plasma cells (Figure 2E and Supplementary Figure 5C). Persistent clones had higher mean mutation frequency (paired *t*‐test, *P* = 4.0 × 10^−7^) (Figure 2F). Together, this suggests the depletion of less mature B cells and persistence of antigen‐experienced cells.

As transitional and naïve B cells are preferentially depleted and subsequently repopulated, we examined the BCRs expressed by these cells. Patients with SLE had shorter CDR3 lengths in both transitional and naïve B cells compared with controls, and this was consistent at pretreatment and late post‐treatment (Kruskal‐Wallis with Dunn's test, adjusted *P* = 0.03) (Figure 2G and H). Patients with SLE also showed biased IGHJ gene usage, particularly in IGHJ3 and IGHJ6 in naïve B cells (Supplementary Figure 5D). Similar signatures were observed in IgM^+^/IgD^+^ SHM^−^ sequences from bulk data (Supplementary Figure 5E and F).

Single‐cell TCR data showed no consistent differences in repertoire characteristics after rituximab (Supplementary Figure 6).

### Transcriptomic changes in non‐B cells after rituximab

**Figure 3:**
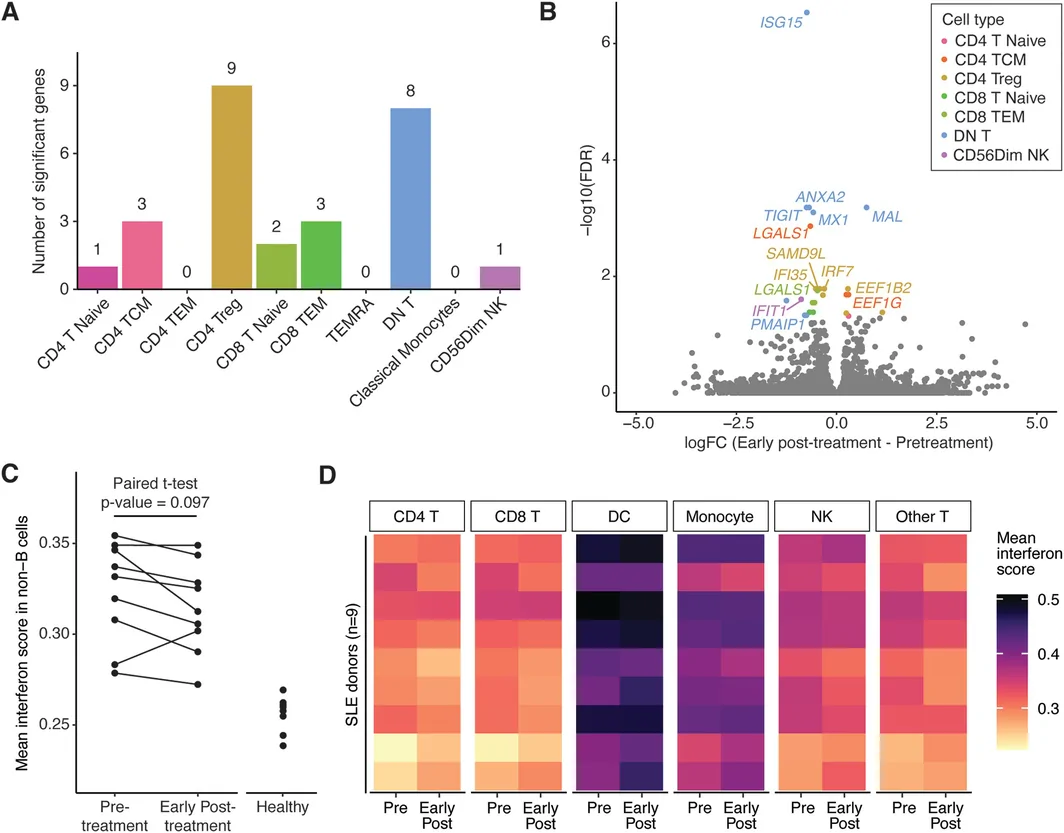
Gene expression changes in non‐B cells following rituximab. (A) Number of significant DEGs in non‐B cells between pretreatment and early post‐treatment (FDR < 0.05, n = 9 vs 9). Only cell types with at least 10 cells in all patients at both time points were included in the analysis. (B) DEGs between early post‐treatment and pretreatment. Statistically significant results are colored by cell type. (C) Mean interferon score of non‐B cells at pretreatment and early post‐treatment to rituximab. Interferon score was calculated using genes from the Reactome gene set “Interferon signaling" (R‐HSA‐913531). Healthy control scores are shown as reference. (D) Mean interferon score of each non‐B cell subtype at pretreatment and early post‐treatment by patient. DEG, differentially expressed gene; FDR, false discovery rate. Color figure can be viewed in the online issue, which is available at http://onlinelibrary.wiley.com/doi/10.1002/art.70116/abstract.

Given that rituximab may lead to change in other immune cells, we analyzed transcriptional changes following rituximab in non‐B cells. We performed differential gene expression analysis in non‐B cell subtypes with more than 10 cells in all patients at each time point and compared pretreatment with early post‐treatment cells to identify early changes after rituximab. A total of 27 genes (26 unique) were identified across 7 of 10 immune cell subtypes (Figure 3A and B). Regulatory CD4 T (Treg) cells and DN T cells had the most DEGs with nine and eight genes, respectively.

Given the known role of interferon signaling in inflammation, autoantibody production, and disease prognosis in SLE,^29^ we focused on transcriptomic changes in interferon‐related genes. Out of 26 DEGs, 8 genes were within the “interferon signaling” pathway (Reactome pathway R‐HSA‐913531): IFI35, XAF1, and IRF7 in Treg cells; IFI44 in effector memory CD8 T cells; USP18 in naïve CD8 T cells; IFIT1 in CD56Dim NK cells; and ISG15 and MX1 in DN T cells. For most, expression decreased following treatment (Supplementary Figure 7A). To assess overall pathway activity, we calculated a score for each cell using “interferon signaling pathway” genes from Reactome (R‐HSA‐913531) (see “Methods” section). No consistent changes in interferon pathway scores were observed in non‐B cells globally or at the cell‐type level (Figure 3C and D, Supplementary Figure 7B).

### B cell depletion and repopulation in rituximab responders and nonresponders

**Figure 4:**
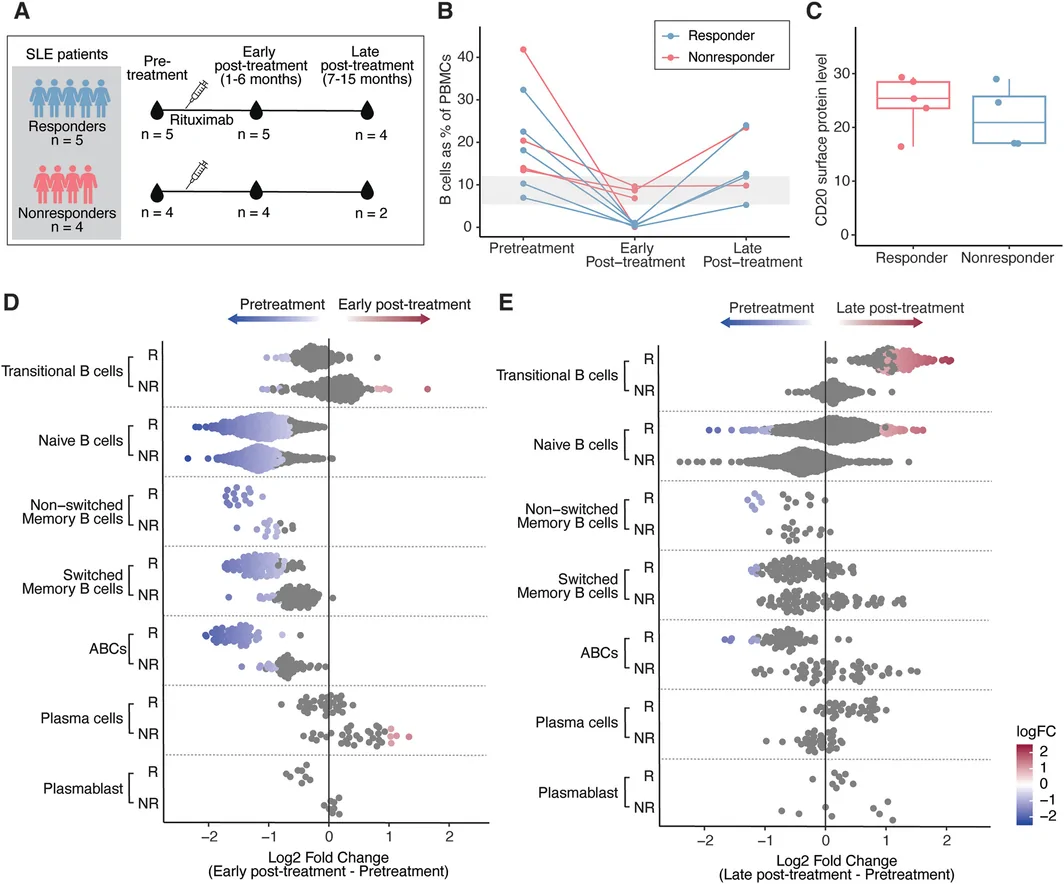
B cell depletion and repopulation in rituximab responders and nonresponders. (A) Patients with SLE stratified into responders (n = 5) and nonresponders (n = 4) based on clinical response to rituximab. Early post‐treatment samples were collected for all patients, whereas late post‐treatment samples were available for four of five responders and two of four nonresponders. (B) Proportion of B cells among total PBMCs at pretreatment, early post‐treatment, and late post‐treatment, as measured by single‐cell sequencing. The shaded region represents the range observed in healthy controls (n = 8). (C) Average CD20 surface protein expression in B cells from rituximab responders and nonresponders at pretreatment. (D) and (E) Differential B cell abundance in rituximab responders (R) and nonresponders (NR). (D) Comparison between pretreatment and early post‐treatment; (E) comparison between pretreatment and late post‐treatment. Each point represents a cell neighborhood predominantly composed of the annotated B cell subtype. Neighborhoods with significant changes in abundance between time points within each response group are colored by the log_2_FC. Gray points indicate neighborhoods without significant change. PBMCs, peripheral blood mononuclear cells; SLE, systemic lupus erythematosus. Color figure can be viewed in the online issue, which is available at http://onlinelibrary.wiley.com/doi/10.1002/art.70116/abstract.

To assess potential cell‐type–level differences based on the clinical response, we examined cell‐type abundance over time in rituximab responders and nonresponders separately (Figure 4A). Response was assessed 12 months post‐treatment (Supplementary Methods), with five of nine patients responding to rituximab.

To quantify overall B cell depletion, we first calculated the proportion of B cells in single‐cell PBMC data pre‐ and post‐treatment (Figure 4B). These proportions correlated well with matched flow cytometry data (Supplementary Figure 8A and B) (Pearson R = 0.98, *P* value <2.2 × 10^−16^). The B cell proportion was higher in patients with SLE than in healthy controls at pretreatment (Wilcoxon test *P* value = 0.0037), but no difference existed between responders and nonresponders (Wilcoxon test *P* value >0.05). All patients showed reduced B cell proportions after treatment, but incomplete depletion (>1% B cells at early post‐treatment) was observed in three of four nonresponders, with an average of 8.4% of PBMCs remaining as B cells. This incomplete depletion was not due to differences in CD20 surface protein levels at pretreatment (*t*‐test *P* value >0.05) (Figure 4C and Supplementary Figure 8C). By late post‐treatment, B cell proportions were not significantly different from pretreatment (repeated measures analysis of variance *P* value >0.05) (Figure 4B).

To determine the difference between responders and nonresponders in B cell depletion, we performed differential cell abundance analysis between pretreatment and early post‐treatment within each group (Figure 4D). This revealed significant depletion in ABCs and switched memory B cells in responders that was not found in nonresponders, suggesting a difference in the effectiveness of depletion in these cells. Naïve B cells and non‐switched memory B cells depleted similarly, whereas nonresponders showed a slight increase in plasma cells.

We then compared late post‐treatment with pretreatment to understand B cell repopulation (Figure 4E). Responders maintained low ABC and switched memory B cell levels, whereas transitional B cells rapidly repopulated. Consistent with these changes, BCR data showed IgM usage predominating at late post‐treatment (Supplementary Figure 9A). There were no consistent differences in Shannon entropy, probability of generation, or mean mutation frequency between responders and nonresponders across time points (Supplementary Figure 9B–D). In nonresponders, no significant differences in abundance were observed between pretreatment and late post‐treatment. However, there was a trend toward enrichment of IgA and increased persistent IgA clones, suggesting the survival of antigen‐experienced cells (Supplementary Figure 9E).

### Transcriptomic changes in repopulated naïve B cells in responders

Following rituximab, substantial turnover in B cell populations occurred in responders. To investigate transcriptomic differences between depleted and repopulated B cells, we performed differential gene expression analysis between pretreatment and late post‐treatment in responders. We focused specifically on naïve B cells because these were completely depleted at early post‐treatment (Supplementary Figure 10), and all patients had at least 10 cells at both time points. Other B cell subtypes had insufficient cell numbers and were therefore excluded.

**Figure 5:**
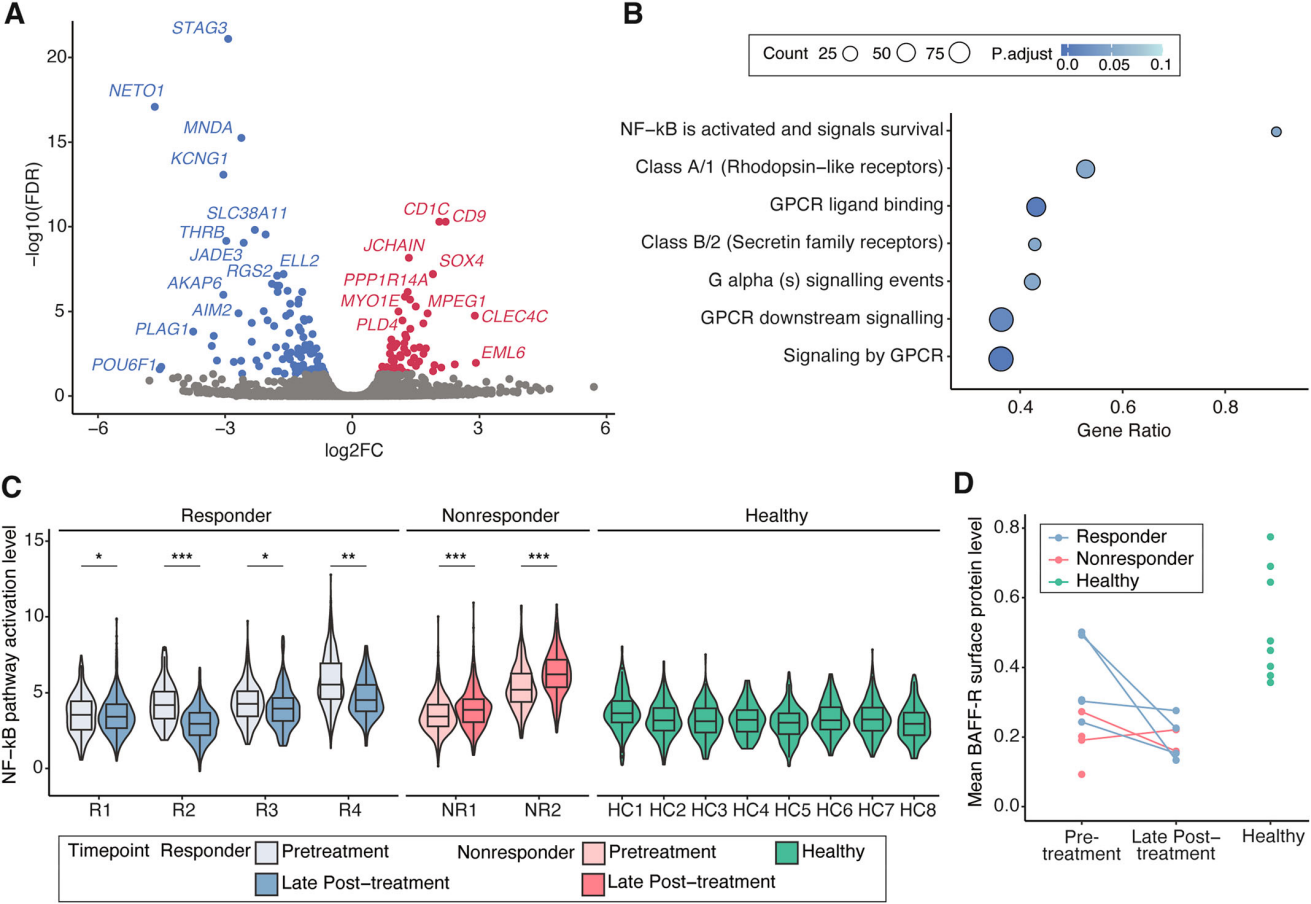
The transcriptomic profile of repopulated B cells in rituximab responders. (A) Volcano plot showing DEGs (n = 171, FDR < 0.05) in naïve B cells from rituximab responders, comparing pretreatment with late post‐treatment. Each point represents a gene, with significance and log_2_FC plotted on the *y*‐ and *x*‐axes, respectively. (B) Top Reactome pathways enriched among DEGs in responder naïve B cells between pretreatment and late post‐treatment. Gene ratio indicates the proportion of core enrichment genes relative to the total number of genes within each pathway. (C) NF‐κB pathway activation scores in naïve B cells from individual patients at pretreatment and late post‐treatment. Violin plots show the distribution of activation scores across cells, whereas box plots indicate the IQR (box), median (center line), and minimum/maximum values excluding outliers (whiskers). Differences in pathway activation between time points were assessed per patient using the Wilcoxon rank‐sum test. Adjusted *P* values: * < 0.05; ** < 0.01; *** < 0.001. (D) Mean BAFF‐R cell surface protein levels on naïve B cells from healthy controls and patients with SLE at pretreatment and late post‐treatment, measured by CITE‐seq using antibody‐derived tags. BAFF‐R, B cell activating factor receptor; CITE‐seq, cellular indexing of transcriptomes and epitopes by sequencing; DEG, differentially expressed gene; FDR, false discovery rate; NF‐κB, nuclear factor kappa‐light‐chain‐enhancer of activated B cells; SLE, systemic lupus erythematosus. Color figure can be viewed in the online issue, which is available at http://onlinelibrary.wiley.com/doi/10.1002/art.70116/abstract.

A total of 171 genes (60 up‐regulated and 111 down‐regulated) showed significant changes (FDR <0.05) (Figure 5A and Supplementary Table 3). Many genes, including *SOCS1*, *SOCS3*, *PTGER4*, *CD83*, *PRDM1*, and *AIF1*, were related to the regulation of immune processes and have previously been reported in SLE genome‐wide association studies (GWAS Catalog^30^: trait “systemic lupus erythematosus”). Pathway analysis further highlighted NF‐κB pathway and G protein‐coupled receptor signaling (Figure 5B).

Given the central role of NF‐κB signaling in immune regulation, we sought to quantify its activity at the single‐cell level. We calculated the NF‐κB pathway activation level of individual cells (Supplementary Methods). In all responders, the level of NF‐κB pathway activation was significantly lower in late post‐treatment naïve B cells compared with pretreatment (Wilcoxon rank‐sum test *P* value <0.05), resembling levels in healthy controls (Figure 5C). Nonresponders had higher activation levels at late post‐treatment. A similar pattern was also observed in memory B cell subtypes, including ABCs (Supplementary Figure 11A).

BAFF‐R, when in contact with BAFF, activates the NF‐κB pathway.^31^ Hence, BAFF‐R surface protein levels on naïve B cells were investigated to assess whether changes in receptor expression could be linked to altered NF‐κB pathway activity (Figure 5D). BAFF‐R levels were significantly lower at late post‐treatment compared with pretreatment in three of four responders (Wilcoxon rank‐sum test *P* value <0.001) (Supplementary Figure 11B), aligning with the change in NF‐κB pathway activation.

### Gene expression changes interacting with rituximab response in non‐B cells

Broader immune responses may differ between responders and nonresponders following rituximab. To investigate this, we analyzed gene expression changes in non‐B cells by comparing response‐specific effects from pretreatment with early post‐treatment (see “Methods” section). For instance, this approach identifies genes that increase in expression in responders but decrease in nonresponders.

**Figure 6:**
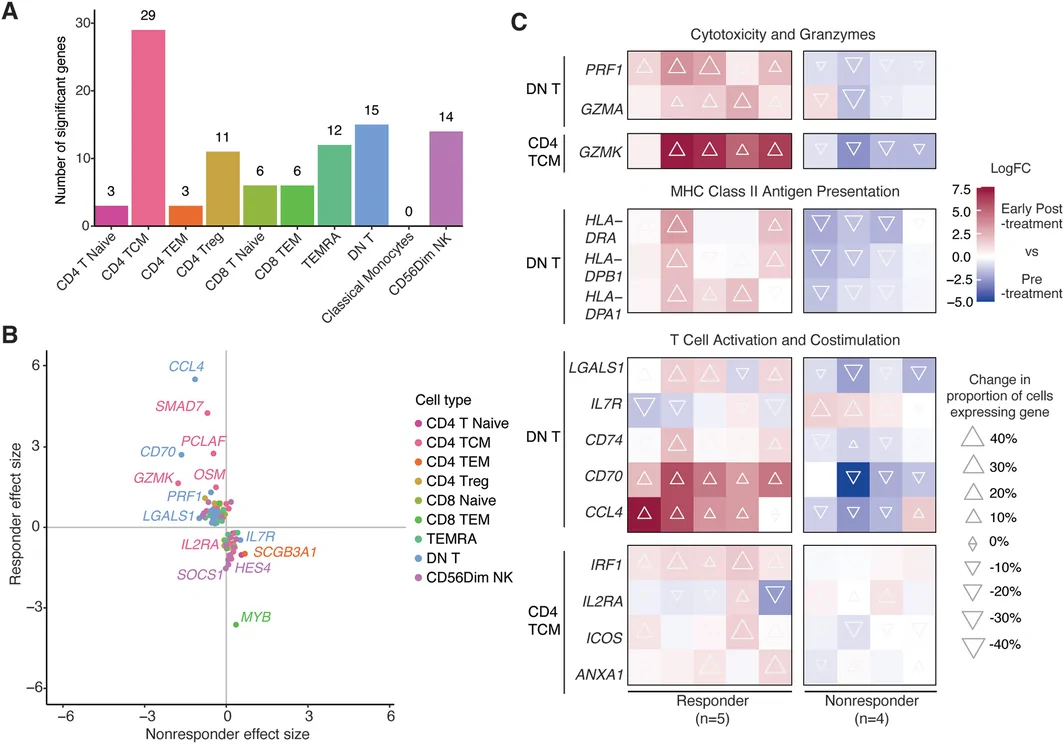
Gene expression changes interacting with rituximab response in non‐B cells. (A) The number of significant genes in non‐B cell subtypes where rituximab response status alters gene expression changes between pretreatment and early post‐treatment (FDR < 0.05). Only cell types with at least 10 cells in all patients at both time points were analyzed. (B) Effect sizes of significant genes where response status modifies gene expression changes between pretreatment and early post‐treatment. The *x*‐axis shows the effect size in nonresponders, and the *y*‐axis shows the effect size in responders. Each point represents a gene and is colored by the immune cell subtype in which it was significantly altered. (C) Significant genes in DN T cells and CD4 TCM cells that function in “cytotoxicity and granzymes”, “MHC class II antigen presentation”, and “T cell activation and costimulation.” The fold changes in expression are shown as color, and changes in the proportion of cells expressing the gene are shown in triangle size and direction. Log_2_FC was calculated from TMM normalized expression levels. Expression values were not adjusted for covariates that were included in the model for testing differential gene expression (patient and cellular detection rate). Gene function annotation was informed by Gene Ontology. DN, double negative; FDR, false discovery rate; MHC, major histocompatibility complex; TCM, CD4 T central memory; TMM, trimmed mean of M values. Color figure can be viewed in the online issue, which is available at http://onlinelibrary.wiley.com/doi/10.1002/art.70116/abstract.

A total of 99 genes (72 unique) in nine immune cell subtypes showed differential expression changes based on rituximab response (FDR <0.05) (Figure 6A and Supplementary Table 4). Among these, CD4 TCMs and DN T cells had the highest number of significant genes, with 29 and 15, respectively. From permutation analysis, this is greater than expected by chance (empirical *P* value <0.05) (Supplementary Figure 12).

Of the 99 genes, 63 showed a greater, positive effect size in responders, whereas nonresponders showed smaller, negative changes (Figure 6B). Many DEGs were involved in cell cytotoxicity (*PRF1*, *GZMA*, *GZMK*), major histocompatibility complex class II antigen presentation (*HLA‐DRA*, *HLA‐DPB1*, *HLA‐DPA1*), and T cell activation (*LGALS1*, *IL7R*, *CD74*, *CD70*, *CCL4*, *IRF1*, *IL2RA*, *ICOS*, *ANXA1*). Most of these genes had increased expression and a higher proportion of expressing cells in responders (Figure 6C). This suggested distinct transcriptomic changes in non‐B cells depending on clinical response.

## DISCUSSION

Through single‐cell profiling, our longitudinal cohort revealed changes in immune cell dynamics following rituximab treatment in SLE. Rituximab led to significant early depletion of naïve and memory B cells while partially sparing antigen‐experienced cells, especially in nonresponders. This was followed by repopulation by naïve and transitional B cells. This aligns with the changes previously reported by flow cytometry in Iwata et al.^32^, ^33^

Rituximab also affected the BCR repertoire, with sequences at early post‐treatment being more mutated, class‐switched, and clonally expanded and with lower generation probabilities, as previously reported.^13^, ^34^ This contrasts with changes in the repertoire observed after CD19 CAR‐T cell therapy, in which IgG and IgA expressing cells are almost completely depleted, and sequences post‐treatment have higher generation probabilities.^12^ The proportion of naïve B cells increased following CD19 CAR‐T cell therapy^35^ but decreased after rituximab treatment in both studies, which may explain some differences. Persistent B cell clones were also identified, which were enriched for mature memory and antibody secreting cells. This expands on previous findings in patients with SLE treated with rituximab,^13^ and these clones share similar features to persistent clones identified in patients with myasthenia gravis receiving rituximab.^36^ Such persistent clones could be a target for future studies to understand their association with treatment response, clinical manifestations, and autoantibody profiles.

The repertoire perturbations we observed are primarily driven by depletion and subsequent repopulation of transitional and naïve B cells, which represent the largest fraction of B cells. Their loss immediately after rituximab enriches for mutated, class‐switched sequences among the surviving antibody‐producing cells, whereas their later repopulation restores repertoire diversity and generation probability toward baseline. In contrast, changes in smaller subsets such as ABCs and switched memory B cells may not be fully reflected in repertoire‐wide diversity metrics. Overall, changes in the repertoire were not sustained over time, returning to baseline by late post‐treatment.

We observed differences in IGHJ usage and CDR3 length between pretreatment patients with SLE and healthy controls that were specific to naïve and transitional cells (Figure 4F and G). Biased V(D)J usage, including for IGHJ genes, has been previously reported in SLE.^37^ Certain V genes, including IGHV4‐34, have also been implicated in SLE pathology,^38^ but we found no significant differences in these genes. Both longer and shorter CDR3 lengths have also been reported in SLE.^13^, ^39^, ^40^ Long CDR3 regions are associated with autoreactivity and are normally selected against during development.^41^ These conflicting findings may be because of the cell populations sampled, with shorter CDR3 being reported in total B cells or naïve B cells, as in this study.

A small number of genes exhibited differential expression in non‐B cells post‐rituximab. Certain interferon pathway genes decreased in Treg, naïve CD8 T, effector memory CD8 T, DN T, and CD56Dim NK cells, but not globally. This contrasts with the broad decrease in interferon pathway genes in non‐B cells after CAR‐T cell mediated B cell depletion.^12^ This may reflect differences in target cells (CD20^+^ vs CD19^+^) because antibody secreting cells (CD20^−^CD19^+^ plasma cells and plasmablasts) remaining after rituximab could activate the interferon pathway in non‐B cells. Furthermore, CAR‐T therapy may lead to B cell depletion in lymphoid organs, thus affecting a broader range of immune cells. It has also been associated with a greater down‐regulation of Type I and II interferon responses across all PBMCs than that observed in whole blood following standard pharmacotherapy, including rituximab.^35^

The modest gene expression changes observed in non‐B cells may also reflect divergent changes between responders and nonresponders, which could mask underlying effects in the pooled analysis. Indeed, our response‐interaction analysis revealed more DEGs (99 vs 27 genes), suggesting that many transcriptional changes in non‐B cells are response‐associated rather than universal. Finally, the use of a conservative pseudobulk framework and stringent significance thresholds may have reduced sensitivity to subtle effects, particularly given the cohort size.

Using single‐cell data, we were able to explore specific memory B cell subtypes, especially those that partially persisted through depletion, including ABCs. ABCs can contribute to autoimmune diseases by developing through TLR7/9 stimulation and producing autoantibodies and inflammatory cytokines.^42^, ^43^ In SLE, ABC frequency correlates with disease activity,^42^, ^44^ and their deletion in MRL.lpr lupus‐prone mice suppresses disease.^45^ A previous study demonstrated a significant reduction of ABCs (CD11c^+^CD21^−^) following rituximab.^46^ In our study, ABCs were fully depleted in responders and remained lowly abundant for an extended period even when overall B cell abundance returned to pretreatment levels. In nonresponders, we saw incomplete depletion in two of four patients. This indicates that a fraction of antigen‐experienced cells can persist despite rituximab, reflecting differences in localization or accessibility of B cell subsets. As a disease‐relevant cell type, further studies will be needed to clarify how the dynamics of ABC depletion contribute to response to rituximab in SLE.

In our study, patients who responded to rituximab showed increased abundance of transitional B cells at late post‐treatment, whereas the memory B cell compartment remained lowly abundant. This finding aligns with previous research characterizing B cell repopulation after rituximab, in which patients achieving full depletion had delayed repopulation and a B cell compartment primarily consisting of transitional B cells at 55 weeks.^47^ Transitional B cells are immature precursors of naïve B cells and may play a regulatory role through the production of interleukin (IL)‐10 and other cytokines.^48^ Therefore, this alteration in the balance between transitional B cells and memory B cells may reflect the response to rituximab. However, transitional B cells producing high levels of proinflammatory cytokines, such as IL‐6,^49^ have been associated with disease activity in SLE and could potentially contribute to relapse.

In non‐B cell populations, several genes showed response‐dependent transcriptional changes following rituximab. Genes associated with T cell activation and cytotoxicity were up‐regulated in responder CD4 CTMs and DN T cells. In SLE, DN T cells are known to be expanded and contribute to disease pathology by promoting autoantibody production, secreting cytokines such as IL‐4, IL‐17, and IFN‐γ, and infiltrating target organs like the kidney.^50^, ^51^ DN T cells have also been reported to regulate B cell apoptosis and inhibit B cell proliferation and plasma cell differentiation.^52^ Our analysis identified increased expression of *LGALS1* in responder DN T cells and decreased expression in nonresponders. This gene regulates B cell apoptosis and is linked to rituximab resistance in non‐Hodgkin lymphoma.^53^ These suggest that DN T cells may contribute to B cell apoptosis during rituximab‐induced depletion.

Limitations to our study include the sample size and time points. A larger cohort would enable more comprehensive comparisons between responders and nonresponders at each time point, including cell type proportions and gene expression, while further adjusting for demographic and clinical heterogeneity. Additional time points would provide insight into specific response trajectories.

By conducting longitudinal single‐cell sequencing, our study provides valuable insights into cellular changes from rituximab‐induced B cell depletion and highlights the role of various immune cells in SLE pathogenesis.

## AUTHOR CONTRIBUTIONS

All authors contributed to at least one of the following manuscript preparation roles: conceptualization AND/OR methodology, software, investigation, formal analysis, data curation, visualization, and validation AND drafting or reviewing/editing the final draft. As corresponding author, Drs Botto and Davenport confirm that all authors have provided the final approval of the version to be published and take responsibility for the affirmations regarding article submission (eg, not under consideration by another journal), the integrity of the data presented, and the statements regarding compliance with institutional review board/Declaration of Helsinki requirements.

## Supporting information


**Disclosure Form**:

**Supplementary Figure 1.** Flowchart of the single‐cell data quality control (QC) process, showing the number of droplets removed at each filtering step due to low quality. A total of 169,513 cells passed QC and were included in downstream analysis.
**Supplementary Figure 2.** Cell type markers used for immune cell annotation. **a**) Marker genes from scRNA‐seq data used to annotate immune cell subtypes. Gene expression levels were normalized and log‐transformed using Scanpy's default settings. **b**) Surface protein markers from CITE‐seq data.
**Supplementary Figure 3. a**) Constant region gene usage from BCR data **b**) BCR heavy chain mutation frequency **c**) BCR light chain mutation frequency.
**Supplementary Table 1.** Participant demographics.
**Supplementary Table 2.** Summary of clinical information at each sample collection timepoint for SLE patients.
**Supplementary Figure 4.** CD20 surface protein levels on immune cell types in PBMC measured by CITE‐seq.
**Supplementary Figure 5. a**) Shannon Entropy of bulk repertoire against B cell % of PBMC from single cell data, both at early post‐treatment. Higher values of Shannon Entropy indicate greater diversity. **b**) Numbers of unique and overlapping clones identified within each SLE patient across timepoints. **c**) B cell UMAP as shown in Fig. 2e highlighting cells with sequences belonging to persistent clones, split across timepoints. **d**) IGHJ gene usage in naïve B cells at pretreatment, late post‐treatment and in healthy controls. Dunn's test was used for pairwise comparisons in genes with an FDR‐adjusted p‐value <0.05 from Kruskal‐Wallis test. FDR‐adjusted p‐value *< 0.05. **e**) Mean IgH CDR3 length in IgM+/IgD+ SHM‐ sequences from bulk repertoire data. FDR adjusted p‐value, Kruskal‐Wallis followed by Dunn's test. **f**) IGHJ gene usage in IgM+/IgD+ SHM‐ sequences from bulk repertoire data at pretreatment, late post‐treatment and in healthy controls. FDR‐adjusted p‐value *< 0.05 **< 0.01.
**Supplementary Figure 6. a**) Shannon Entropy of single cell TCR repertoire across timepoints. All samples were subset to 451 cells across 1000 iterations and a mean was calculated. **b**) Mean generation probability across timepoints, calculated by OLGA on nucleotide CDR3 sequence. Higher generation probability indicates a sequence that is more likely to occur by chance. **c**) Heatmap of TRBV gene usage across individuals and timepoints.
**Supplementary Figure 7. a**) Gene expression levels of interferon pathway genes that are significantly differentially expressed between pretreatment and early post‐treatment in SLE patients. Gene expression levels were TMM normalised. **b**) Violin plot of interferon activity score in non‐B cells from each patient and timepoint. Interferon score was calculated using genes from the Reactome gene set ‘Interferon signaling (R‐HSA‐913531)’.
**Supplementary Figure 8. a**) Proportion of B cells of each patient measured by flow cytometry at pretreatment, early post‐treatment, and late post‐treatment. The shaded region represents the range observed in healthy controls (n=8). **b**) Correlation between B cell percentage calculated from single cell data (out of PBMC) and flow cytometry data (out of CD45+ cells). The dotted line represents the x=y line. **c**) CD20 surface protein levels of each B cell subtype measured by CITE‐seq at pretreatment in responders, non‐responders and healthy controls. No significant differences were observed between groups with ANOVA (p‐value > 0.05).
**Supplementary Figure 9. a**) Isotype usage in total B cells from single cell data, split by timepoint and rituximab response. **b**) Shannon entropy of bulk BCR repertoire samples across timepoints coloured by treatment response. Higher values indicate greater diversity. All samples were subset to 1,616 UMIs across 1,000 iterations and a mean was calculated. **c**) Mean generation probability of bulk BCR repertoire samples across timepoints, calculated by OLGA on nucleotide CDR3 sequence. Higher generation probability indicates a sequence that is more likely to occur by chance. **d**) Mean mutation frequency of bulk BCR repertoire samples across timepoints coloured by treatment response. Samples are coloured by responder status. **e**) Proportion of repertoire occupied by each isotype split between sequences forming persistent or non‐persistent clones in responders and non‐responders.
**Supplementary Figure 10.** The proportion of each B cell subtype out of all PBMC across timepoints by patient. scRNA‐seq data was used to calculate cell type proportions. The shaded region shows the range of healthy controls. Pre = pretreatment; Early = early post‐treatment; Late = late post‐treatment.
**Supplementary Table 3.** DEGs between pretreatment and late post‐treatment naive B cells in responders. (FDR < 0.05, CPM = counts per million).
**Supplementary Figure 11. a**) Heatmap of mean NF‐kB pathway activation score of B cell subtypes at pretreatment and late post‐treatment. Score was calculated using decoupleR with PROGENy pathway gene weights and were scaled around zero. **b**) BAFFR cell surface protein levels on naive B cells. Two‐sample Wilcoxon tests done between pretreatment and late post‐treatment cells within each patient. *p‐value < 0.05 **p‐value < 0.01 ***p‐value < 0.001.
**Supplementary Table 4.** DEGs between pretreatment and early post‐treatment non‐B cells that change differently between responders and non‐responders. (CPM = counts per million).
**Supplementary Figure 12.** Distribution of the number of significant genes from permutation analyses in non‐B cells (FDR < 0.05). Genes in which response status alters the expression change between pretreatment and early post‐treatment were tested while the response label for each patient was shuffled to all possible response permutations. The true result was considered significant if it had an empirical p‐value < 0.05.

**Data S1.** Supplementary Methods.
