# Supplementary material for: Single‐Cell Level Characterization of B Cell Depletion and Repopulation Following Rituximab in Systemic Lupus Erythematosus
Source: Arthritis Rheumatol. 2026 Apr 20;78(9):1907–19. doi: 10.1002/art.70116 (PMC13507573; doi:10.1002/art.70116)
Supplement: Supplementary file 1 [file ART-78-1907-s003.pdf]

ICMJE DISCLOSURE FORM

Date:___1 December 2025____
Your Name:__Haerin Jang____
Manuscript Title:__Single-cell level characterization of B cell depletion and repopulation following rituximab in systemic lupus erythematosus______________________________
Manuscript number (if known):___ ar-25-0984 ____

In the interest of transparency, we ask you to disclose all relationships/activities/interests listed below that are related to the content of your manuscript. "Related" means any relation with for-profit or not-for-profit third parties whose interests may be affected by the content of the manuscript. Disclosure represents a commitment to transparency and does not necessarily indicate a bias. If you are in doubt about whether to list a relationship/activity/interest, it is preferable that you do so.

The following questions apply to the author's relationships/activities/interests as they relate to the current manuscript only.

The author's relationships/activities/interests should be defined broadly. For example, if your manuscript pertains to the epidemiology of hypertension, you should declare all relationships with manufacturers of antihypertensive medication, even if that medication is not mentioned in the manuscript.

In item #1 below, report all support for the work reported in this manuscript without time limit. For all other items, the time frame for disclosure is the past 36 months.

		**Name all entities with whom you have this relationship or indicate none (add rows as needed)**	**Specifications/Comments (e.g., if payments were made to you or to your institution)**
		**Time frame: Since the initial planning of the work**	
1	All support for the present manuscript (e.g., funding, provision of study materials, medical writing, article processing charges, etc.) **No time limit for this item.**	Wellcome Trust	The Wellcome Sanger Institute is funded by the Wellcome Trust [220540/Z/20/A]
		**Time frame: past 36 months**	
2	Grants or contracts from any entity (if not indicated in item #1 above).	X None	
3	Royalties or licenses	X None	
4	Consulting fees	X None	

5	Payment or honoraria for lectures, presentations, speakers bureaus, manuscript writing or educational events	X____None	
6	Payment for expert testimony	X____None	
7	Support for attending meetings and/or travel	X____None	
8	Patents planned, issued or pending	X____None	
9	Participation on a Data Safety Monitoring Board or Advisory Board	____None	
10	Leadership or fiduciary role in other board, society, committee or advocacy group, paid or unpaid	X____None	
11	Stock or stock options	X____None	
12	Receipt of equipment, materials, drugs, medical writing, gifts or other services	X____None	
13	Other financial or non-financial interests	X____None	

Please place an "X" next to the following statement to indicate your agreement:

__X_ I certify that I have answered every question and have not altered the wording of any of the questions on this form.

ICMJE DISCLOSURE FORM

Date:_____05/12/2025_______________________________________
Your Name:___NORZAWANI BUANG @ M YASSIN___________________________
Manuscript Title:__Single-cell level characterization of B cell depletion and repopulation following rituximab in systemic lupus erythematosus_____________________________
Manuscript number (if known):___ ar-25-0984 ____________________________

In the interest of transparency, we ask you to disclose all relationships/activities/interests listed below that are related to the content of your manuscript. "Related" means any relation with for-profit or not-for-profit third parties whose interests may be affected by the content of the manuscript. Disclosure represents a commitment to transparency and does not necessarily indicate a bias. If you are in doubt about whether to list a relationship/activity/interest, it is preferable that you do so.

The following questions apply to the author's relationships/activities/interests as they relate to the current manuscript only.

The author's relationships/activities/interests should be defined broadly. For example, if your manuscript pertains to the epidemiology of hypertension, you should declare all relationships with manufacturers of antihypertensive medication, even if that medication is not mentioned in the manuscript.

In item #1 below, report all support for the work reported in this manuscript without time limit. For all other items, the time frame for disclosure is the past 36 months.

		**Name all entities with whom you have this relationship or indicate none (add rows as needed)**	**Specifications/Comments (e.g., if payments were made to you or to your institution)**
**Time frame: Since the initial planning of the work**			
1	All support for the present manuscript (e.g., funding, provision of study materials, medical writing, article processing charges, etc.) **No time limit for this item.**	X____None	
**Time frame: past 36 months**			
2	Grants or contracts from any entity (if not indicated in item #1 above).	X____None	
3	Royalties or licenses	X____None	
4	Consulting fees	X____None	

5	Payment or honoraria for lectures, presentations, speakers bureaus, manuscript writing or educational events	X None	
6	Payment for expert testimony	X None	
7	Support for attending meetings and/or travel	X None	
8	Patents planned, issued or pending	X None	
9	Participation on a Data Safety Monitoring Board or Advisory Board	X None	
10	Leadership or fiduciary role in other board, society, committee or advocacy group, paid or unpaid	X None	
11	Stock or stock options	X None	
12	Receipt of equipment, materials, drugs, medical writing, gifts or other services	X None	
13	Other financial or non-financial interests	X None	

Please place an "X" next to the following statement to indicate your agreement:

_X_ I certify that I have answered every question and have not altered the wording of any of the questions on this form.

ICMJE DISCLOSURE FORM

Date: 01/12/25
Your Name: Catherine Sutherland
Manuscript Title:__Single-cell level characterization of B cell depletion and repopulation following rituximab in systemic lupus erythematosus______________________________
Manuscript number (if known):___ ar-25-0984**____**

In the interest of transparency, we ask you to disclose all relationships/activities/interests listed below that are related to the content of your manuscript. "Related" means any relation with for-profit or not-for-profit third parties whose interests may be affected by the content of the manuscript. Disclosure represents a commitment to transparency and does not necessarily indicate a bias. If you are in doubt about whether to list a relationship/activity/interest, it is preferable that you do so.

The following questions apply to the author's relationships/activities/interests as they relate to the current manuscript only.

The author's relationships/activities/interests should be defined broadly. For example, if your manuscript pertains to the epidemiology of hypertension, you should declare all relationships with manufacturers of antihypertensive medication, even if that medication is not mentioned in the manuscript.

In item #1 below, report all support for the work reported in this manuscript without time limit. For all other items, the time frame for disclosure is the past 36 months.

		**Name all entities with whom you have this relationship or indicate none (add rows as needed)**	**Specifications/Comments (e.g., if payments were made to you or to your institution)**
**Time frame: Since the initial planning of the work**			
1	All support for the present manuscript (e.g., funding, provision of study materials, medical writing, article processing charges, etc.) **No time limit for this item.**	The Wellcome Trust	This research was funded in part by the Wellcome Trust [220540/Z/20/A]
		Lupus Research Alliance	Salary support from Lupus Research Alliance Global Team Science award
**Time frame: past 36 months**			
2	Grants or contracts from any entity (if not indicated in item #1 above).	X___None	
3	Royalties or licenses	X___None	

4	Consulting fees	X None	
5	Payment or honoraria for lectures, presentations, speakers bureaus, manuscript writing or educational events	X None	
6	Payment for expert testimony	X None	
7	Support for attending meetings and/or travel	X None	
8	Patents planned, issued or pending	X None	
9	Participation on a Data Safety Monitoring Board or Advisory Board	X None	
10	Leadership or fiduciary role in other board, society, committee or advocacy group, paid or unpaid	X None	
11	Stock or stock options	X None	
12	Receipt of equipment, materials, drugs, medical writing, gifts or other services	X None	
13	Other financial or non-financial interests	X None	

Please place an "X" next to the following statement to indicate your agreement:

X I certify that I have answered every question and have not altered the wording of any of the questions on this form.

ICMJE DISCLOSURE FORM

Date:_____2025/12/08____
Your Name:____Wanseon Lee____
Manuscript Title:__ Single-cell level characterization of B cell depletion and repopulation following rituximab in systemic lupus erythematosus______________________________
Manuscript number (if known):___ ar-25-0984 ____

In the interest of transparency, we ask you to disclose all relationships/activities/interests listed below that are related to the content of your manuscript. "Related" means any relation with for-profit or not-for-profit third parties whose interests may be affected by the content of the manuscript. Disclosure represents a commitment to transparency and does not necessarily indicate a bias. If you are in doubt about whether to list a relationship/activity/interest, it is preferable that you do so.

The following questions apply to the author's relationships/activities/interests as they relate to the current manuscript only.

The author's relationships/activities/interests should be defined broadly. For example, if your manuscript pertains to the epidemiology of hypertension, you should declare all relationships with manufacturers of antihypertensive medication, even if that medication is not mentioned in the manuscript.

In item #1 below, report all support for the work reported in this manuscript without time limit. For all other items, the time frame for disclosure is the past 36 months.

		**Name all entities with whom you have this relationship or indicate none (add rows as needed)**	**Specifications/Comments (e.g., if payments were made to you or to your institution)**
**Time frame: Since the initial planning of the work**			
1	All support for the present manuscript (e.g., funding, provision of study materials, medical writing, article processing charges, etc.) **No time limit for this item.**	__**X**__None	
**Time frame: past 36 months**			
2	Grants or contracts from any entity (if not indicated in item #1 above).	__**X**__None	
3	Royalties or licenses	__**X**__None	
4	Consulting fees	__**X**__None	

5	Payment or honoraria for lectures, presentations, speakers bureaus, manuscript writing or educational events	__**X**__None	
6	Payment for expert testimony	__**X**__None	
7	Support for attending meetings and/or travel	__**X**__None	
8	Patents planned, issued or pending	__**X**__None	
9	Participation on a Data Safety Monitoring Board or Advisory Board	__**X**__None	
10	Leadership or fiduciary role in other board, society, committee or advocacy group, paid or unpaid	__**X**__None	
11	Stock or stock options	__**X**__None	
12	Receipt of equipment, materials, drugs, medical writing, gifts or other services	__**X**__None	
13	Other financial or non-financial interests	__**X**__None	

Please place an "X" next to the following statement to indicate your agreement:

_X_ I certify that I have answered every question and have not altered the wording of any of the questions on this form.

ICMJE DISCLOSURE FORM

Date:_____11/12/2025____
Your Name:__Lauren Overend____
Manuscript Title:__Single-cell level characterization of B cell depletion and repopulation following rituximab in systemic lupus erythematosus________________________________
Manuscript number (if known):____

In the interest of transparency, we ask you to disclose all relationships/activities/interests listed below that are related to the content of your manuscript. "Related" means any relation with for-profit or not-for-profit third parties whose interests may be affected by the content of the manuscript. Disclosure represents a commitment to transparency and does not necessarily indicate a bias. If you are in doubt about whether to list a relationship/activity/interest, it is preferable that you do so.

The following questions apply to the author's relationships/activities/interests as they relate to the current manuscript only.

The author's relationships/activities/interests should be defined broadly. For example, if your manuscript pertains to the epidemiology of hypertension, you should declare all relationships with manufacturers of antihypertensive medication, even if that medication is not mentioned in the manuscript.

In item #1 below, report all support for the work reported in this manuscript without time limit. For all other items, the time frame for disclosure is the past 36 months.

		**Name all entities with whom you have this relationship or indicate none (add rows as needed)**	**Specifications/Comments (e.g., if payments were made to you or to your institution)**
**Time frame: Since the initial planning of the work**			
1	All support for the present manuscript (e.g., funding, provision of study materials, medical writing, article processing charges, etc.) **No time limit for this item.**	The Wellcome Trust Centre for Human Genetics	My PhD was funded by the Genomic Medicine and Statistics Program at the Wellcome Trust Centre for Human Genetics, Oxford.
**Time frame: past 36 months**			
2	Grants or contracts from any entity (if not indicated in item #1 above).	X None	
3	Royalties or licenses	X None	

4	Consulting fees	X None	
5	Payment or honoraria for lectures, presentations, speakers bureaus, manuscript writing or educational events	X None	
6	Payment for expert testimony	X None	
7	Support for attending meetings and/or travel	X None	
8	Patents planned, issued or pending	X None	
9	Participation on a Data Safety Monitoring Board or Advisory Board	X None	
10	Leadership or fiduciary role in other board, society, committee or advocacy group, paid or unpaid	X None	
11	Stock or stock options	X None	
12	Receipt of equipment, materials, drugs, medical writing, gifts or other services	X None	
13	Other financial or non-financial interests	X None	

Please place an "X" next to the following statement to indicate your agreement:

_X__ I certify that I have answered every question and have not altered the wording of any of the questions on this form.

ICMJE DISCLOSURE FORM

Date:_09/12/2025
Your Name: Tarran Singh Rupall
Manuscript Title:__Single-cell level characterization of B cell depletion and repopulation following rituximab in systemic lupus erythematosus______________________________
Manuscript number (if known):___ ar-25-0984____

In the interest of transparency, we ask you to disclose all relationships/activities/interests listed below that are related to the content of your manuscript. "Related" means any relation with for-profit or not-for-profit third parties whose interests may be affected by the content of the manuscript. Disclosure represents a commitment to transparency and does not necessarily indicate a bias. If you are in doubt about whether to list a relationship/activity/interest, it is preferable that you do so.

The following questions apply to the author's relationships/activities/interests as they relate to the current manuscript only.

The author's relationships/activities/interests should be defined broadly. For example, if your manuscript pertains to the epidemiology of hypertension, you should declare all relationships with manufacturers of antihypertensive medication, even if that medication is not mentioned in the manuscript.

In item #1 below, report all support for the work reported in this manuscript without time limit. For all other items, the time frame for disclosure is the past 36 months.

		**Name all entities with whom you have this relationship or indicate none (add rows as needed)**	**Specifications/Comments (e.g., if payments were made to you or to your institution)**
		**Time frame: Since the initial planning of the work**	
1	All support for the present manuscript (e.g., funding, provision of study materials, medical writing, article processing charges, etc.) **No time limit for this item.**	X____None	
		**Time frame: past 36 months**	
2	Grants or contracts from any entity (if not indicated in item #1 above).	X____None	
3	Royalties or licenses	X____None	
4	Consulting fees	X____None	

5	Payment or honoraria for lectures, presentations, speakers bureaus, manuscript writing or educational events	X___None	
6	Payment for expert testimony	X___None	
7	Support for attending meetings and/or travel	X___None	
8	Patents planned, issued or pending	X___None	
9	Participation on a Data Safety Monitoring Board or Advisory Board	X___None	
10	Leadership or fiduciary role in other board, society, committee or advocacy group, paid or unpaid	X___None	
11	Stock or stock options	X___None	
12	Receipt of equipment, materials, drugs, medical writing, gifts or other services	X___None	
13	Other financial or non-financial interests	X___None	

Please place an "X" next to the following statement to indicate your agreement:

X I certify that I have answered every question and have not altered the wording of any of the questions on this form.

ICMJE DISCLOSURE FORM

Date:__1/12/25____
Your Name:__Katie Burnham____
Manuscript Title:__Single-cell level characterization of B cell depletion and repopulation following rituximab in systemic lupus erythematosus______________________________
Manuscript number (if known):____

In the interest of transparency, we ask you to disclose all relationships/activities/interests listed below that are related to the content of your manuscript. "Related" means any relation with for-profit or not-for-profit third parties whose interests may be affected by the content of the manuscript. Disclosure represents a commitment to transparency and does not necessarily indicate a bias. If you are in doubt about whether to list a relationship/activity/interest, it is preferable that you do so.

The following questions apply to the author's relationships/activities/interests as they relate to the current manuscript only.

The author's relationships/activities/interests should be defined broadly. For example, if your manuscript pertains to the epidemiology of hypertension, you should declare all relationships with manufacturers of antihypertensive medication, even if that medication is not mentioned in the manuscript.

In item #1 below, report all support for the work reported in this manuscript without time limit. For all other items, the time frame for disclosure is the past 36 months.

		**Name all entities with whom you have this relationship or indicate none (add rows as needed)**	**Specifications/Comments (e.g., if payments were made to you or to your institution)**
		**Time frame: Since the initial planning of the work**	
1	All support for the present manuscript (e.g., funding, provision of study materials, medical writing, article processing charges, etc.) **No time limit for this item.**	Wellcome Trust	The Wellcome Sanger Institute is funded by the Wellcome Trust [220540/Z/20/A]
		**Time frame: past 36 months**	
2	Grants or contracts from any entity (if not indicated in item #1 above).	X None	
3	Royalties or licenses	X None	
4	Consulting fees	X None	

5	Payment or honoraria for lectures, presentations, speakers bureaus, manuscript writing or educational events	X___None	
6	Payment for expert testimony	X___None	
7	Support for attending meetings and/or travel	X___None	
8	Patents planned, issued or pending	X___None	
9	Participation on a Data Safety Monitoring Board or Advisory Board	X___None	
10	Leadership or fiduciary role in other board, society, committee or advocacy group, paid or unpaid	X___None	
11	Stock or stock options	X___None	
12	Receipt of equipment, materials, drugs, medical writing, gifts or other services	X___None	
13	Other financial or non-financial interests	X___None	

Please place an "X" next to the following statement to indicate your agreement:

X I certify that I have answered every question and have not altered the wording of any of the questions on this form.

ICMJE DISCLOSURE FORM

Date:___________01/DEC/2025____
Your Name:_________MATTHEW PICKERING____
Manuscript Title:__Single-cell level characterization of B cell depletion and repopulation following rituximab in systemic lupus erythematosus_____________________________
Manuscript number (if known):___ ar-25-0984 ____

In the interest of transparency, we ask you to disclose all relationships/activities/interests listed below that are related to the content of your manuscript. "Related" means any relation with for-profit or not-for-profit third parties whose interests may be affected by the content of the manuscript. Disclosure represents a commitment to transparency and does not necessarily indicate a bias. If you are in doubt about whether to list a relationship/activity/interest, it is preferable that you do so.

The following questions apply to the author's relationships/activities/interests as they relate to the current manuscript only.

The author's relationships/activities/interests should be defined broadly. For example, if your manuscript pertains to the epidemiology of hypertension, you should declare all relationships with manufacturers of antihypertensive medication, even if that medication is not mentioned in the manuscript.

In item #1 below, report all support for the work reported in this manuscript without time limit. For all other items, the time frame for disclosure is the past 36 months.

		**Name all entities with whom you have this relationship or indicate none (add rows as needed)**	**Specifications/Comments (e.g., if payments were made to you or to your institution)**
		**Time frame: Since the initial planning of the work**	
1	All support for the present manuscript (e.g., funding, provision of study materials, medical writing, article processing charges, etc.) **No time limit for this item.**	Imperial NIHR BRC funding	
		**Time frame: past 36 months**	
2	Grants or contracts from any entity (if not indicated in item #1 above).	X____None	
3	Royalties or licenses	X____None	
4	Consulting fees	Consulting fees from Alexion, Achillion,	

		Annexon, Apellis, Biocryst, ChemoCentryx, Complement Therapeutics, Gemini, Gyroscope, MIRNA Therapeutics, Ormeros, Q32bio Pharma, and Sobi.	
5	Payment or honoraria for lectures, presentations, speakers bureaus, manuscript writing or educational events	Fees for Educational activities with Sobi and Medscape	
6	Payment for expert testimony	X___None	
7	Support for attending meetings and/or travel	Travel paid for by Sobi to ERA-EDTA 2025 for Sobi Educational activity on C3 glomerulopathy	
8	Patents planned, issued or pending	X___None	
9	Participation on a Data Safety Monitoring Board or Advisory Board	X___None	
10	Leadership or fiduciary role in other board, society, committee or advocacy group, paid or unpaid	X___None	
11	Stock or stock options	X___None	
12	Receipt of equipment, materials, drugs, medical writing, gifts or other services	X___None	
13	Other financial or non-financial interests	X___None	

Please place an "X" next to the following statement to indicate your agreement:

_X__ I certify that I have answered every question and have not altered the wording of any of the questions on this form.

ICMJE DISCLOSURE FORM

Date:____1/12/2025______________________________
Your Name:_______________Marina Botto______________________
Manuscript Title:__Single-cell level characterization of B cell depletion and repopulation following rituximab in systemic lupus erythematosus______________________
Manuscript number (if known):______ ar-25-0984

In the interest of transparency, we ask you to disclose all relationships/activities/interests listed below that are related to the content of your manuscript. "Related" means any relation with for-profit or not-for-profit third parties whose interests may be affected by the content of the manuscript. Disclosure represents a commitment to transparency and does not necessarily indicate a bias. If you are in doubt about whether to list a relationship/activity/interest, it is preferable that you do so.

The following questions apply to the author's relationships/activities/interests as they relate to the current manuscript only.

The author's relationships/activities/interests should be defined broadly. For example, if your manuscript pertains to the epidemiology of hypertension, you should declare all relationships with manufacturers of antihypertensive medication, even if that medication is not mentioned in the manuscript.

In item #1 below, report all support for the work reported in this manuscript without time limit. For all other items, the time frame for disclosure is the past 36 months.

		Name all entities with whom you have this relationship or indicate none (add rows as needed)	Specifications/Comments (e.g., if payments were made to you or to your institution)
		**Time frame: Since the initial planning of the work**	
1	All support for the present manuscript (e.g., funding, provision of study materials, medical writing, article processing charges, etc.) **No time limit for this item.**	X None	
		**Time frame: past 36 months**	
2	Grants or contracts from any entity (if not indicated in item #1 above).	X None	
3	Royalties or licenses	X None	
4	Consulting fees	X None	

5	Payment or honoraria for lectures, presentations, speakers bureaus, manuscript writing or educational events	X___None	
6	Payment for expert testimony	X___None	
7	Support for attending meetings and/or travel	X___None	
8	Patents planned, issued or pending	X___None	
9	Participation on a Data Safety Monitoring Board or Advisory Board	X___None	
10	Leadership or fiduciary role in other board, society, committee or advocacy group, paid or unpaid	X___None	
11	Stock or stock options	X___None	
12	Receipt of equipment, materials, drugs, medical writing, gifts or other services	X___None	
13	Other financial or non-financial interests	X___None	

Please place an "X" next to the following statement to indicate your agreement:

_x__ I certify that I have answered every question and have not altered the wording of any of the questions on this form.

ICMJE DISCLOSURE FORM

Date:_______15/12/2025____
Your Name:____Emma Davenport____
Manuscript Title:__Single-cell level characterization of B cell depletion and repopulation following rituximab in systemic lupus erythematosus______________________________
Manuscript number (if known):___ ar-25-0984____

In the interest of transparency, we ask you to disclose all relationships/activities/interests listed below that are related to the content of your manuscript. "Related" means any relation with for-profit or not-for-profit third parties whose interests may be affected by the content of the manuscript. Disclosure represents a commitment to transparency and does not necessarily indicate a bias. If you are in doubt about whether to list a relationship/activity/interest, it is preferable that you do so.

The following questions apply to the author's relationships/activities/interests as they relate to the current manuscript only.

The author's relationships/activities/interests should be defined broadly. For example, if your manuscript pertains to the epidemiology of hypertension, you should declare all relationships with manufacturers of antihypertensive medication, even if that medication is not mentioned in the manuscript.

In item #1 below, report all support for the work reported in this manuscript without time limit. For all other items, the time frame for disclosure is the past 36 months.

		**Name all entities with whom you have this relationship or indicate none (add rows as needed)**	**Specifications/Comments (e.g., if payments were made to you or to your institution)**
		**Time frame: Since the initial planning of the work**	
1	All support for the present manuscript (e.g., funding, provision of study materials, medical writing, article processing charges, etc.) **No time limit for this item.**	Wellcome	Core funding from Wellcome to my institute to support my salary, PhD students and members of my team as well as consumables funding for data generation.
		**Time frame: past 36 months**	
2	Grants or contracts from any entity (if not indicated in item #1 above).	X None	
3	Royalties or licenses	X None	

4	Consulting fees	X____None	
5	Payment or honoraria for lectures, presentations, speakers bureaus, manuscript writing or educational events	X____None	
6	Payment for expert testimony	X____None	
7	Support for attending meetings and/or travel	X____None	
8	Patents planned, issued or pending	X____None	
9	Participation on a Data Safety Monitoring Board or Advisory Board	X____None	
10	Leadership or fiduciary role in other board, society, committee or advocacy group, paid or unpaid	X____None	
11	Stock or stock options	X____None	
12	Receipt of equipment, materials, drugs, medical writing, gifts or other services	X____None	
13	Other financial or non-financial interests	X____None	

Please place an "X" next to the following statement to indicate your agreement:

_x__ I certify that I have answered every question and have not altered the wording of any of the questions on this form.
